# Completeness of MMR Vaccination and Durability of Vaccine-Induced Antibody Responses in Children with Inflammatory Bowel Disease

**DOI:** 10.3390/biomedicines14071526

**Published:** 2026-07-07

**Authors:** Ivan S. Samolygo, Alexey A. Tinkov, Marina A. Manina, Anton S. Antishin, Albina S. Pestova, Ekaterina A. Yablokova, Ekaterina V. Prutskova, Mikhail P. Kostinov, Svetlana I. Erdes

**Affiliations:** 1 Department of Propaedeutics of Children’s Diseases, N.F. Filatov Clinical Institute of Children’s Health, Sechenov First Moscow State Medical University (Sechenov University), 119991 Moscow, Russia; 2 Laboratory of Molecular Dietetics, Federal State Autonomous Educational Institution of Higher Education Sechenov First Moscow State Medical University (Sechenov University), 119991 Moscow, Russia; 3 Institute of Cellular and Intracellular Symbiosis, Russian Academy of Sciences, 460000 Orenburg, Russia; 4 Department of Children’s Diseases, N.F. Filatov Clinical Institute of Children’s Health, Sechenov First Moscow State Medical University (Sechenov University), 119991 Moscow, Russia; 5 Department of Pediatrics, Ulyanovsk State University, 432017 Ulyanovsk, Russia; 6 Laboratory of Vaccine Prevention and Immunotherapy of Allergic Diseases, Federal State Budgetary Scientific Institution «I. Mechnikov Research Institute of Vaccines and Sera», 105064 Moscow, Russia; 7 Department of Epidemiology and Modern Vaccination Technologies, Federal State Autonomous Educational Institution of Higher Education, Sechenov First Moscow State Medical University (Sechenov University), 119991 Moscow, Russia

**Keywords:** IBD, MMR, vaccination, antibodies

## Abstract

**Background:** Children with IBD are at increased risk of suboptimal maintenance of vaccine-induced immunity, particularly when the MMR vaccination course is incomplete before diagnosis and initiation of immunosuppressive therapy. We conducted a prospective study to evaluate the durability of antibody responses to measles, mumps, and rubella in pediatric IBD patients and to determine how completeness of MMR vaccination influences long-term antibody persistence over 12 months. **Methods:** Sixty children with IBD were included. Demographic characteristics, clinical disease activity (PUCAI/PCDAI), inflammatory markers (CRP, ESR), and fecal calprotectin were extracted from electronic medical records. Vaccination completeness was ascertained from documented immunization history. Serum antibodies to measles, rubella, and mumps were measured at baseline and after 12 months. Seroprotection was defined using standard laboratory thresholds. Antibody decay over time was assessed with paired non-parametric tests, and time to loss of seroprotection was analyzed using Cox proportional hazards models. In addition, Bayesian ANOVA modeling was applied to quantify evidence for differences in antibody concentrations and decay kinetics according to vaccination status. **Results:** Overall, 66.7% of patients had completed the full MMR vaccination course. At baseline, seroprotection rates were 48.3% for measles, 76.7% for rubella, and 70% for mumps. After 12 months, median antibody concentrations declined significantly for all three antigens. Corresponding seroprotection rates changed to 46.7% for measles (*p* = 0.414), 70% for rubella (*p* = 0.046), and 66.7% for mumps (*p* = 0.157). Incomplete MMR vaccination was identified as a major modifiable risk factor for accelerated antibody waning in children with IBD. Cox regression demonstrated that incompletely vaccinated patients had a 2.13-fold higher risk of losing measles seroprotection (95% CI 1.07–4.24; *p* = 0.032), a 5.27-fold higher risk for rubella (95% CI 1.86–14.95; *p* = 0.002), and a 4.82-fold higher risk for mumps (95% Cl 1.68–13.85; *p* = 0.004). Bayesian analyses provided decisive evidence that vaccination completeness strongly influences baseline antibody levels. **Conclusions:** Incomplete MMR vaccination is associated with markedly reduced durability of vaccine-induced immunity to measles, mumps, and rubella in children with IBD. These findings underscore the need for systematic prevaccination screening, timely completion of age-appropriate vaccination before initiation of immunosuppressive therapy when feasible, and individualized serological monitoring to identify patients at highest risk of vaccine-preventable infections.

## 1. Introduction

Over the past decade, the incidence of vaccine-preventable infectious diseases has increased worldwide [1]. The growing prevalence of vaccine hesitancy, the consequences of the COVID-19 pandemic, and the decline in herd immunity have become key contributors to the rising burden of vaccine-preventable infections [1,2]. In the United States, 338 measles cases were reported between 2020 and 2024, despite the country previously being considered measles-free [3]. According to the World Health Organization, more than 300,000 measles cases were recorded globally in 2024, with the majority occurring in developing countries [4,5,6]. Since 2015, Western Europe has also experienced an increase in measles incidence [7]. Undoubtedly, active migration processes in these countries have contributed to the destabilization of the epidemiological situation across the continent [7]. According to official statistics, more than 6627 cases of measles were registered in Russia in 2025, which is 3.4 times lower than in 2024 [8]. The epidemiological situation regarding rubella appears somewhat more favorable [8]. Nevertheless, rubella incidence remains high in developing countries [9]. One of the most illustrative examples is the attempt to eliminate rubella in Japan. Despite high vaccination coverage among the population, increases in rubella incidence were observed during 2012–2013 and 2018–2019 [10]. Detailed outbreak investigations demonstrated that the primary source of infection was adult men who had not undergone revaccination in adulthood, subsequently transmitting the infection to infants under one year of age and adolescents [11]. Since 2017, the Russian Federation has been declared rubella-free. However, according to the latest data, an increase in rubella cases was recorded in 2025, with more than 374 cases nationwide [8]. Mumps currently remains one of the most prevalent vaccine-preventable infections worldwide [12]. One of the reasons for its widespread circulation is the generally mild clinical course observed in most cases. According to the World Health Organization, more than 380,000 mumps cases were reported globally in 2023 [13]. Studies conducted in Europe between 2021 and 2022 demonstrated an increase in mumps incidence among children older than 10 years [14]. A recent systematic review identified peaks in mumps incidence during 2004–2009 and 2016–2020 [12]. Regional analysis revealed higher incidence rates in the Americas (29.2%) and the Eastern Mediterranean region (28.8%) compared with Europe (7.6%) and Southeast Asia (9.6%) [12]. Over the past decade, several large mumps outbreaks have been reported in Russia, particularly during 2017–2019 and 2023–2025 [8]. According to official statistics, 1673 mumps cases were registered in Russia in 2025, with notable regional differences in disease distribution [8]. MMR vaccination remains a highly effective strategy for the prevention of these infections. Following its introduction, the incidence of measles, rubella, and mumps was successfully brought under control worldwide [15]. However, accumulating evidence indicates that vaccine-induced antibody levels inevitably decline even in healthy individuals, while cellular immunity may be insufficient for rapid pathogen elimination. On average, measles-specific antibody titers decrease by 5.6% annually, corresponding to a half-life of approximately 12 years [16]. Crooke S. N. et al. demonstrated an annual decline of 2.2% in rubella-specific antibody levels [17]. Collectively, these factors may explain the increasing incidence of vaccine-preventable infections observed in recent decades, thereby threatening the maintenance of herd immunity. Reduced population immunity poses a substantial risk to the most vulnerable groups, particularly immunocompromised individuals.

In patients with inflammatory bowel disease (IBD), disease onset may occur at an age when routine immunization has not yet been completed [18]. Continuation of vaccination is often challenging, as the measles–mumps–rubella (MMR) vaccine is live-attenuated and its administration is restricted in patients receiving immunosuppressive therapy [19]. As a result, vaccination may be postponed indefinitely. Previous studies have demonstrated that children with IBD frequently fail to complete the recommended measles, mumps, and rubella vaccination schedule [20,21]. Moreover, several reports indicate that vaccine-induced immunity may wane more rapidly in patients undergoing immunosuppressive therapy [22,23]. Our previous study similarly demonstrated suboptimal persistence of measles and rubella antibodies in children with IBD, particularly among those younger than 6 years of age and those who had not completed the full vaccination schedule [24].

In addition to incomplete vaccination and immunosuppressive therapy, other factors, such as dysregulation of immune response mechanisms [25], may also affect the durability of post-vaccination immunity. However, significant gaps remain in understanding the longitudinal persistence of vaccine-induced immunity in relation to vaccination completeness. Therefore, the aim of the present study was to evaluate the levels and dynamics of measles, mumps, and rubella antibodies in children with IBD and to assess factors potentially associated with impaired antibody persistence.

## 2. Materials and Methods

This prospective observational study included 60 pediatric patients with IBD: 41 (68.3%) with Crohn’s disease (CD) and 19 (31.7%) with ulcerative colitis (UC). The diagnosis of IBD was established according to the revised Porto criteria of ESPGHAN (2014) [26]. Patients were enrolled between August 2024 and February 2025. Each patient had blood samples collected at baseline and at 12-month follow-up. The final 12-month follow-up visit was completed in February 2026.

Inclusion criteria:

• Age between 3 years 1 month and 17 years 11 months;
• Documented receipt of at least one dose of the MMR vaccine;
• Low disease activity (PUCAI < 10 for UC; PCDAI < 11 for CD) or moderate disease activity (PUCAI < 35 for UC; PCDAI < 30 for CD);
• Absence of acute respiratory infection at the time of blood sampling;
• Written informed consent obtained from a legal guardian for children under 14 years of age, and from both the patient and legal guardian for those aged ≥14 years.

Exclusion criteria:

• A diagnosis of IBD-unclassified (IBD-U);
• High disease activity (PUCAI > 35; PCDAI > 30);
• A documented history of measles, mumps, or rubella infection;
• A history of emergency vaccination against measles, mumps, or rubella due to epidemiological indications;
• No documented evidence of MMR vaccination administered after 12 months of age;
• Receipt of plasma, intravenous immunoglobulin, or other antibody-containing blood products within the preceding 12 months.

The study was approved by the Local Ethics Committee of I.M. Sechenov First Moscow State Medical University (Sechenov University), Moscow, Russian Federation (protocol No. 18-24, dated 18 July 2024). All patients were evaluated at the Department of Gastroenterology, Clinic of Childhood Diseases, Sechenov University. Written informed consent was obtained from all participants and/or their legal representatives prior to enrollment. Vaccination data were obtained from official immunization records. Patients who had received at least one dose of the MMR vaccine but had not received the second dose were classified as having incomplete vaccination. Those who had received two documented doses were classified as fully vaccinated.

Peripheral venous blood samples were collected twice: at baseline and at 12-month follow-up. Blood was drawn into single-use plastic tubes containing a clot activator. After standing for 15 min at 4 °C to allow clot formation, samples were centrifuged at 3000 rpm for 10 min. The separated serum was aliquoted into sterile single-use microtubes (Eppendorf type), labeled, and stored at −70 °C until analysis. All laboratory analyses were performed at the Laboratory of Vaccine Prevention and Immunotherapy of Allergic Diseases using certified equipment from the Core Facility Center of the I.I. Mechnikov Research Institute of Vaccines and Sera (Moscow, Russia).

Serum IgG antibodies against measles, rubella, and mumps viruses were quantified using commercially available enzyme-linked immunosorbent assay (ELISA) kits (Vector-Best, Novosibirsk, Russia), according to the manufacturer’s instructions [27,28,29]. Seroprotection thresholds for measles, rubella, and mumps were defined according to the manufacturer’s instructions (Vector-Best, Russia).

For measles-specific IgG (Vector-Best Measles IgG ELISA kit (Vector-Best, Novosibirsk, Russia) [27], results were interpreted as follows: antibody concentrations < 0.11 IU/mL were considered negative; ≥0.18 IU/mL concentrations were considered positive. Borderline values (0.12–0.17 IU/mL) were classified as negative, as such levels are not considered fully protective against infection.

For rubella-specific IgG (Vector-Best Rubella IgG ELISA kit (Vector-Best, Novosibirsk, Russia) [28], samples with IgG concentrations < 16 IU/mL were considered negative [28]. For rubella, values > 30 IU/mL were considered protective, as the 16–29 IU/mL range is classified as equivocal by the manufacturer and does not provide robust evidence of protection [28].

Mumps-specific IgG antibodies (Vector-Best Mumps IgG ELISA kit (Vector-Best, Novosibirsk, Russia) [29] were assessed using the Vector-Best Mumps IgG ELISA kit. The results were expressed as optical density (OD) values, and OD values > 0.467 were considered positive according to the manufacturer’s recommendations [29].

To reduce skewness and approximate a normal distribution, antibody concentrations for measles, rubella, and mumps were log10-transformed prior to statistical analysis.

Statistical analyses were performed using Microsoft Excel 2024, jamovi version 2.7.5 (open-source soft-ware, Australia), and RStudio software version 4.5.1. (R foundation for statistical computing, Vienna, Austria). The normality of continuous variables was assessed using the Shapiro–Wilk test. Data with non-normal distribution are presented as medians with interquartile ranges (IQRs), whereas normally distributed data are reported as means with standard deviations (SDs). Categorical variables are presented as counts and percentages. Between-group comparisons for continuous variables were performed using the Mann–Whitney U test for non-normally distributed data. Categorical variables were compared using the Pearson chi-square (χ^2^) test. Odds ratios (ORs) with 95% confidence intervals (95% CIs) were calculated where appropriate. Time-to-event analysis was conducted using Kaplan–Meier curves, with hazard ratios estimated using Cox proportional hazards regression models. Survival analysis was performed using time since last MMR vaccination as the time scale. Patients who were seronegative at baseline were considered to have lost seroprotection at an unknown time prior to study entry. Those who were seropositive at baseline but seronegative at 12 months were considered to have lost seroprotection during the follow-up interval. This approach estimates the median duration of seroprotection based on cross-sectional baseline data combined with 12-month longitudinal follow-up, and it is designed to account for variability in time since last vaccination. Associations between log_10_-transformed antibody concentrations and independent variables (e.g., age) were assessed using linear regression analysis. For paired comparisons, the paired-samples *t*-test was used for continuous variables, and McNemar’s test was applied for categorical variables. Bayesian ANOVA included vaccination completeness (complete vs. incomplete) and time (baseline vs. 12 months) as fixed factors, with participant as a random effect for repeated measures. Default Cauchy priors (scale = 0.707) were used for effect sizes. Bayes factors (BFs) were interpreted as follows:

BF 1–3 = anecdotal evidence;

BF 3–10 = moderate evidence;

BF 10–100 = strong evidence;

BF >100 = decisive evidence.

All tests were two-sided, and a *p*-value < 0.05 was considered statistically significant.

## 3. Results

### 3.1. Patient Characteristics

Demographic data, disease characteristics, and treatment information were obtained from electronic medical records (Table 1). No significant differences were observed between patients with CD and UC in terms of age (*p* = 0.457) or sex distribution (OR 0.485, 95% Cl 0.154–1.52; *p* = 0.211). The age at disease onset was also comparable between groups (*p* = 0.197). The majority of patients developed IBD during the prepubertal or pubertal period. Disease activity was assessed using the PUCAI and the PCDAI. At study entry, median clinical activity was within the moderate range in both groups: 17.5 (IQR 12.5–20) in the CD group and 15 (IQR 10–20) in the UC group. Extraintestinal manifestations were significantly more frequent in patients with CD compared to those with UC (39% vs. 5.3%, OR 12.0, 95% Cl 1.45–99.0; *p* = 0.006). Unfavorable disease phenotypes were more commonly observed in CD than in UC (14.6% vs. 5.3%), although this difference did not reach statistical significance (OR 0.315, 95% Cl 0.0351–2.82; *p* = 0.28). At baseline, laboratory markers indicated moderate inflammatory activity. No significant differences were found between groups in C-reactive protein levels (*p* = 0.44) or the erythrocyte sedimentation rate (*p* = 0.500). The median fecal calprotectin level was 189 µg/g (IQR 112–218) in patients with UC and 202 µg/g (IQR 133–235) in patients with CD.

At study entry, patients were receiving various treatment regimens. Among patients with UC, 31.6% were receiving mesalazine monotherapy, compared with 17% of patients with CD. Thiopurine therapy (azathioprine) for at least 6 months had been administered to 15.8% of patients with UC and 29.3% of patients with CD. Biologic therapy with anti-TNF-α agents for at least 6 months was being received by 52.4% of patients with UC and 53.7% of patients with CD.

### 3.2. Characteristics of Patients with IBD Depending on the Completeness of MMR Vaccination

Baseline characteristics according to vaccination completeness are presented in Table 2. Incompletely vaccinated patients were younger at study entry (*p* = 0.011) and had earlier disease onset (*p* < 0.001). However, time since last MMR dose did not differ significantly between groups (*p* = 0.760). Baseline antibody concentrations for measles, rubella, and mumps were significantly lower in incompletely vaccinated patients (*p* < 0.05).

### 3.3. Vaccination Status and Seroprotection in Patients with IBD

**Figure 1:**
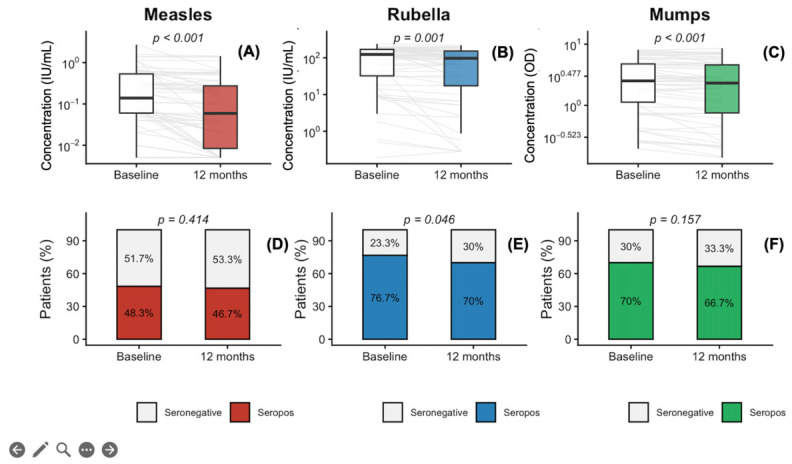
Baseline antibody concentrations (IU/mL) and seroprotection rates (%) against measles, rubella, and mumps and their changes at 12-month follow-up in patients with IBD (**A**–**C**). Antibody titers were log10-transformed prior to analysis and compared using the paired-samples *t*-test. (**D**–**F**) Changes in seroprotection rates were assessed using McNemar’s test.

Overall, 66.7% of patients with IBD had completed the two-dose MMR vaccination schedule. At baseline, the proportion of patients with seroprotective antibody levels was 48.3% for measles, 76.7% for rubella, and 70% for mumps (Figure 1). Linear regression analysis was performed to assess the association between patient age and log10-transformed antibody concentrations. Age was not significantly associated with antibody levels against measles (R^2^ = 0.016, F = 0.925, *p* = 0.34), rubella (R^2^ = 0.012, F = 0.685, *p* = 0.411), or mumps (R^2^ = 0.0053, F = 0.0015, *p* = 0.96) in patients with IBD.

### 3.4. Antibody Levels and Seroprotection at 12-Month Follow-Up

At 12 months after baseline assessment, repeat serum samples were obtained from each patient to determine IgG antibody levels against measles, rubella, and mumps. Antibody concentrations at follow-up were compared with baseline values. Paired analysis of log10-transformed antibody concentrations demonstrated a statistically significant decline in measles-, rubella-, and mumps-specific IgG levels over the 12-month period. The dynamics of antibody titers for measles, rubella, and mumps are presented in Figure 1.

A significant decline in median measles-specific IgG concentrations was observed over 12 months (Figure 1A), falling from 0.14 IU/mL (IQR 0.06–0.531) at baseline to 0.059 IU/mL (IQR 0.0075–0.273) at follow-up (*p* < 0.001). Rubella-specific IgG levels (Figure 1B) decreased from a median of 124 IU/mL (IQR 32.3–169) to 99.3 IU/mL (IQR 17.3–169) (*p* = 0.001). Similarly, mumps-specific IgG concentrations (Figure 1C) declined from 1.2 OD (IQR 0.454–2.25) to 1.08 OD (IQR 0.351–2.14) over the 12-month period (*p* < 0.001). At 12 months, seroprotective measles antibody levels were detected in 46.7% of patients with IBD (Figure 1D), with no significant difference compared to baseline (*p* = 0.414). Seroprotection against rubella was observed in 70% of patients at follow-up (Figure 1E), representing a statistically significant decline compared to baseline (*p* = 0.046). Protective mumps antibody levels were detected in 66.7% of patients at 12 months; however, the difference compared with baseline values was not statistically significant (*p* = 0.157) (Figure 1F).

### 3.5. Antibody Persistence According to Vaccination Completeness and Time Since Vaccination

**Figure 2:**
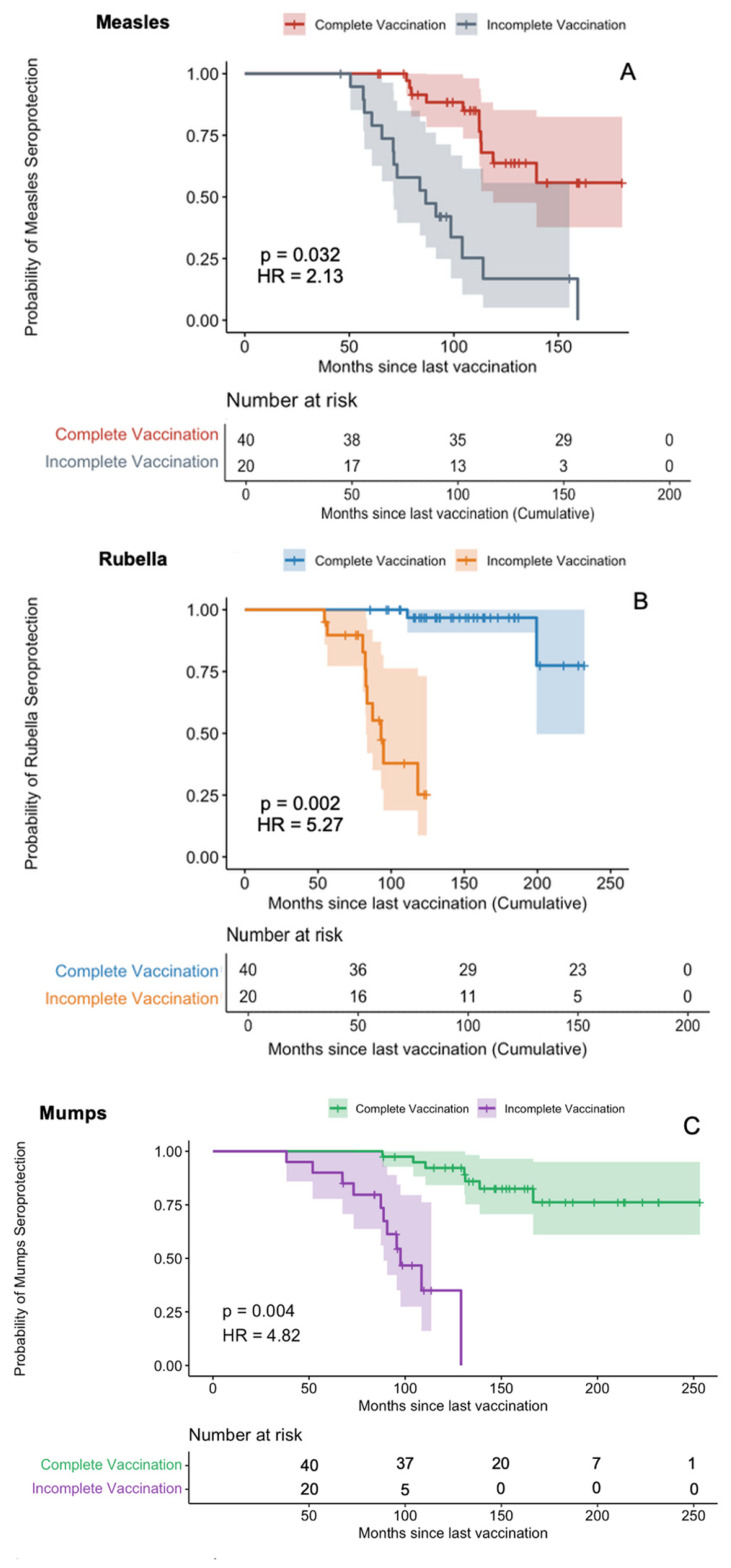
Duration of seroprotection against measles (**A**), rubella (**B**), and mumps (**C**) in children with IBD, stratified by completeness of MMR vaccination. Differences between groups were assessed using the log-rank test. Hazard ratios were estimated using Cox proportional hazards regression analysis.

To assess the long-term persistence of measles, rubella, and mumps antibodies, Kaplan–Meier survival analysis was performed according to vaccination completeness and time elapsed since the last MMR dose (Figure 2). The median duration of measles seroprotection among fully vaccinated patients was 136 months (95% Cl 121–not reached), which was longer than in patients with incomplete vaccination, in whom the median duration of seroprotection was 109 months (95% Cl 98–128). Univariable Cox proportional hazards regression analysis demonstrated that incomplete vaccination was a significant risk factor for earlier loss of protective measles antibody levels. Patients with incomplete vaccination had a twofold higher risk of losing measles seroprotection compared with fully vaccinated patients (HR 2.13; 95% Cl 1.07–4.24; *p* = 0.032) (Figure 2A).

Survival analysis demonstrated a significant effect of complete vaccination on the persistence of rubella antibodies (Figure 2B). The median duration of rubella seroprotection among patients with incomplete vaccination was 123 months, whereas the median was not reached during the study period in fully vaccinated patients. Cox proportional hazards regression analysis showed that the risk of developing a non-protective rubella antibody level was more than fivefold higher in patients with incomplete vaccination compared with those who had completed the full vaccination schedule (HR 5.27; 95% Cl 1.86–14.95; *p* = 0.002) (Figure 2B).

The median duration of mumps seroprotection among patients with incomplete vaccination was 123 months, whereas the median was not reached during the study period in fully vaccinated patients. Cox proportional hazards regression analysis further demonstrated that incomplete vaccination was associated with an almost fourfold increased risk of loss of protective mumps antibody levels (HR 4.82; 95% Cl 1.68–13.85; *p* = 0.004) (Figure 2C).

### 3.6. Changes in Antibody Titers According to Vaccination Completeness at 12-Month Follow-Up

**Figure 3:**
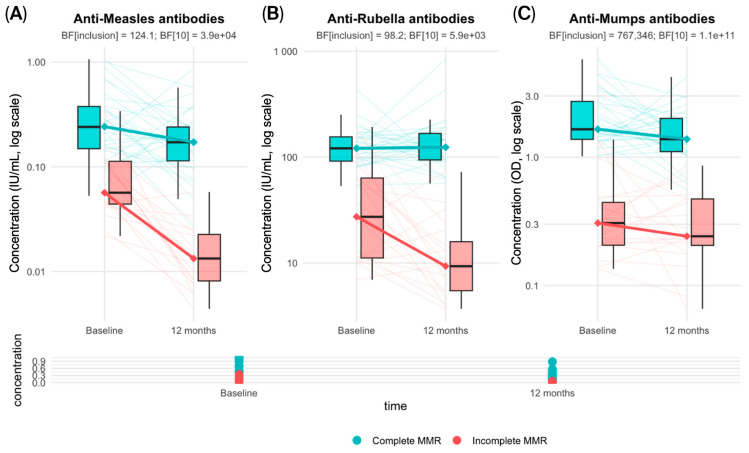
Measles (**A**), rubella (**B**), and mumps (**C**) antibody levels at baseline and at 12-month follow-up according to MMR vaccination completeness in patients with IBD. Antibody concentrations were log10-transformed for analysis (**A**–**C**). Between-group differences were assessed using the Mann–Whitney U test, and longitudinal changes were evaluated using paired-samples *t*-tests. Bayesian ANOVA modeling was performed to assess the effect of vaccination completeness over time.

We further evaluated changes in antibody concentrations according to MMR vaccination completeness (Figure 3). Significant differences were observed between fully and partially vaccinated patients for all three antibodies at both time points. For measles (Figure 3A), the baseline median IgG concentration in fully vaccinated patients was 0.255 IU/mL (IQR 0.085–0.607), compared with 0.06 IU/mL (IQR 0.01–0.09) in partially vaccinated patients (*p* < 0.001). After 12 months, the corresponding values were 0.17 IU/mL (IQR 0.0395–0.336) and 0.01 IU/mL (IQR 0.001–0.0363), respectively (*p* < 0.001). Paired comparisons demonstrated significant within-group declines over time (*p* < 0.001). Bayesian ANOVA modeling indicated strong evidence for an effect of vaccination completeness on measles antibody concentrations (BF_inclusion_ = 124.1). The decline in antibody levels over time occurred in parallel in both groups, with slightly greater reduction in partially vaccinated patients (BF_inclusion_ = 2.21). Post hoc analysis confirmed pronounced differences between fully and partially vaccinated patients (BF_10_ = 38,561).

For rubella (Figure 3B), baseline median IgG concentrations were 145 IU/mL (IQR 85.5–170) in fully vaccinated patients and 35.9 IU/mL (IQR 8.68–153) in partially vaccinated patients (*p* < 0.001). At 12 months, median concentrations were 120 IU/mL (IQR 78.3–160) and 12 IU/mL (IQR 2.22–73.8), respectively (*p* < 0.001). Paired comparisons showed significant reductions over time (*p* = 0.001). Bayesian modeling demonstrated a pronounced effect of vaccination completeness on baseline rubella antibody levels (BF_inclusion_ = 98.2) and on their longitudinal dynamics (BF_inclusion_ = 11.7). Post hoc analysis confirmed strong evidence of differences between groups (BF_10_ = 5942).

For mumps (Figure 3C), baseline median optical density (OD) values were 1.93 (IQR 1.07–2.69) in fully vaccinated patients and 0.348 (IQR 0.151–0.788) in partially vaccinated patients (*p* = 0.008). At 12 months, median OD values were 1.34 (IQR 0.999–2.54) and 0.211 (IQR 0.102–0.72), respectively (*p* = 0.001). Paired comparisons revealed significant within-group declines over time (*p* < 0.001). Bayesian ANOVA further confirmed a significant effect of vaccination completeness on mumps antibody levels (BF_inclusion_ = 767,346). The 12-month follow-up demonstrated a marked decline in mumps antibodies among partially vaccinated patients (BF_inclusion_ = 123). Post hoc analysis provided decisive evidence for differences between fully and partially vaccinated groups (BF_10_ = 1.07 × 10^11^).

## 4. Discussion

Incomplete vaccination remains a common issue among children with chronic systemic diseases, including IBD. In Russia, the second dose of the MMR vaccine is routinely administered at 6–7 years of age [8]. Although antibody waning over time is well documented in healthy populations [17,30], we did not observe a significant association between patient age and antibody concentrations in our cohort. This suggests that vaccination completeness and immunosuppressive therapy exposure may exert stronger effects on antibody persistence than chronological age alone in children with IBD. According to Makarova et al., 66.3% of patients with IBD completed the MMR vaccination schedule [31], which is consistent with the 66.7% completion rate observed in our cohort. In Canada, where the timing of MMR revaccination is comparable to that in Russia, reported MMR completion rates reach 86% among patients with IBD [32]. Notably, even in countries where the age of revaccination is lower, vaccination coverage does not consistently reach target levels. For example, studies conducted in France report MMR revaccination rates ranging from 78% to 91% [20,21]. Several factors may explain the suboptimal vaccination coverage observed in children with IBD. One major limitation is the use of immunosuppressive therapy. Most international guidelines recommend avoiding live-attenuated vaccines, including MMR, in children receiving immunosuppressive treatment, particularly those on high-dose corticosteroids, anti-tumor necrosis factor (anti-TNF) agents, interleukin-12/23 or interleukin-23 inhibitors, or combination therapy with cytotoxic agents [19,33]. Although vaccination prior to initiation of immunosuppressive therapy is recommended, this strategy is not always feasible in clinical practice [19,33]. Surveys among gastroenterologists and pediatricians indicate that fewer than 30% routinely assess vaccination status in ambulatory patients with IBD [34]. Furthermore, approximately 35% of physicians report concerns regarding potential adverse events or disease exacerbation following vaccination, which may influence decisions regarding continuation of immunization [35]. Ultimately, vaccination decisions often depend on parental consent. Parental vaccine hesitancy represents another major barrier to adequate immunization coverage in children with IBD. Studies demonstrate limited awareness among parents regarding the possibility and safety of continuing vaccination after IBD diagnosis [35]. According to Erdes et al., only 22% of parents vaccinated their children after disease onset, whereas 78% declined further immunization [35]. Similar findings were reported by Huth et al., with 19% of participants refusing routine vaccination due to fear of complications [36]. The predominant parental concerns include the risk of disease exacerbation (reported in 3–40% of respondents), fear of vaccine-related adverse events, and doubts about vaccine quality (3–5%) [36,37]. Interestingly, parental attitudes toward vaccination appear to change markedly after disease onset. Prior to IBD diagnosis, more than 90% of parents reportedly supported routine immunization; however, following diagnosis, 78% declined further vaccination, and only 22% proceeded with immunization [35]. This discrepancy between initially favorable attitudes and subsequent refusal suggests the development of ‘acquired vaccine hesitancy’. Collectively, these findings suggest that parents of children with IBD are not fundamentally opposed to vaccination but may avoid it due to concerns about safety and insufficient access to reliable information. In the absence of clear reassurance from healthcare professionals, vaccination may be perceived as an unnecessary or unjustified risk.

In our previous study, incomplete vaccination was identified as a risk factor for lower measles- and rubella-specific antibody concentrations in children with IBD [24]. The present study extends these findings by demonstrating that vaccine-induced antibodies against measles, rubella, and mumps decline more rapidly in patients who had not completed the full vaccination schedule prior to disease onset. Over a 12-month period, the median measles IgG concentration decreased from 0.14 to 0.059 IU/mL (*p* < 0.001), rubella IgG from 124 to 99.3 IU/mL (*p* < 0.001), and mumps antibody levels from 0.324 to 0.253 optical density units (*p* < 0.001). At baseline, only 48.3% of patients with IBD had protective measles antibody levels (defined as >0.18 IU/mL). After 12 months, this proportion declined slightly to 46.7%, although the difference was not statistically significant (*p* = 0.414). Nevertheless, the overall rate of measles seroprotection was notably low. Studies in healthy populations have shown that a single dose of measles vaccine provides suboptimal immunogenicity [38]. Primary vaccine failure is a well-documented phenomenon, with approximately 2–10% of recipients failing to seroconvert after a single MMR dose [38]. In our cohort, 33.3% of patients had incomplete vaccination (received only one dose), which substantially increases the risk of inadequate immune response. Waning immunity is another critical factor. Studies in healthy populations have demonstrated that measles-specific antibody levels decline by approximately 5.6% annually, with a median half-life of 12 years [16]. In our cohort, the median time since last MMR dose was 99 months (approximately 8 years), providing ample time for antibody decay, particularly in patients who received only one dose. Altered immune memory in the context of chronic intestinal inflammation may also play a role. IBD is characterized by dysregulated immune responses, including T-cell exhaustion, impaired T-follicular-helper-cell differentiation, and disruption of B-cell memory compartments [39,40,41]. These immune alterations may compromise the durability of vaccine-induced immunity independently of exogenous immunosuppression. The timing of vaccination relative to disease onset is another potentially important factor. In our cohort, incomplete vaccination was associated with significantly earlier disease onset (median 6.5 years vs. 11 years in fully vaccinated patients, *p* < 0.001; Table 2). This suggests that some children may have developed IBD shortly after receiving their first MMR dose (typically administered at 12 months of age), potentially interfering with antibody maturation or recall responses before the second dose could be administered. Assay-specific thresholds may also influence seroprotection rates. The Vector-Best ELISA uses a protective threshold of >0.18 IU/mL, which is conservative and designed to ensure robust protection. Finally, the country-specific vaccine schedule in Russia (first dose at 12 months, second dose at 6–7 years) is comparable to schedules in many high-income countries and is unlikely to explain the low seroprotection rate. However, the relatively long interval between doses may allow for waning immunity in children who develop IBD during this window, especially if immunosuppressive therapy is started before the second dose and prevents the continuation of vaccination. However, we intentionally applied the manufacturer’s recommended threshold to ensure clinical relevance. Importantly, in our cohort, 25% of fully vaccinated patients lacked protective measles antibody titers. deBruyn et al. reported that measles antibodies were the least persistent among children with IBD, with a seroprotection rate of 60% [23]. Other data are shown by Luana Cagol et al., where high titers of protective measles antibodies were found in patients with IBD after two vaccinations [42]. Similarly, Makarova et al. detected protective measles titers in only 47.7% of pediatric patients with IBD [31], closely aligning with our findings. In contrast, rubella antibodies demonstrated better persistence. At baseline, 76.7% of patients had protective rubella antibody levels, which decreased to 70% at 12 months (*p* = 0.046). This level of seroprotection may be considered relatively satisfactory in the context of pediatric IBD. Comparable results were reported by deBruyn et al., who observed a rubella seroprotection rate of 75% among patients with IBD [23]. Makarova et al. similarly reported protective rubella titers in 74.4% of children with IBD [31]. For mumps, protective antibody levels were detected in 70% of patients at baseline and in 66.7% at 12 months (*p* = 0.157). Previous studies have reported moderate persistence of mumps antibodies in 49.6% to 75.3% of pediatric patients with IBD [31,43], which is consistent with our observations.

Particular attention should be paid to antibody persistence in relation to vaccination completeness. In the context of increasing interest in the phenomenon of accelerated waning of vaccine-induced immunity in patients with IBD, our study confirms the importance of incomplete vaccination as a dominant and modifiable risk factor. We found that measles antibodies exhibited the lowest persistence, even among fully vaccinated patients, with a median duration of seroprotection of 136 months (approximately 11 years), whereas the median duration was not reached for rubella or mumps antibodies. Similar findings were reported by Kostik et al., who demonstrated reduced persistence of measles antibodies compared with other vaccine-induced antibodies in children with juvenile idiopathic arthritis, with incomplete vaccination identified as one of the contributing factors [43]. In our cohort, incomplete vaccination increased the risk of early loss of measles and mumps antibodies by approximately twofold and fivefold, respectively, consistent with previous reports [43]. Bayesian ANOVA modeling provided strong evidence that vaccination completeness exerts a dominant effect on antibody concentrations. Notably, we observed parallel kinetics of measles antibody decline in fully and partially vaccinated groups (BF_inclusion_ = 2.21), suggesting that once low antibody levels are established, the rate of decline may proceed similarly regardless of vaccination status. However, the critical difference lies in baseline antibody concentrations: fully vaccinated patients had a significantly higher median baseline measles IgG level (0.255 IU/mL) compared with partially vaccinated patients (0.06 IU/mL; *p* < 0.001). This difference in starting point implies that, even with comparable rates of decline, partially vaccinated patients reach a non-protective threshold substantially earlier. This observation was further supported by survival analysis, which demonstrated a longer median duration of measles seroprotection in fully vaccinated patients (136 months) compared with partially vaccinated patients (109 months). For rubella antibodies, modeling demonstrated that incomplete vaccination influenced both baseline differences (BF_inclusion_ = 98.2) and the rate of decline over time (BF_inclusion_ = 11.7). The median duration of rubella seroprotection in partially vaccinated patients was 123 months, whereas this threshold was not reached in fully vaccinated patients during the study period. These findings may be particularly relevant for female patients with IBD who are planning future pregnancies, given the well-established risk of severe fetal complications associated with rubella infection during pregnancy [11]. Mumps antibodies showed the most pronounced differences between fully and partially vaccinated groups (BF_10_ = 1.07 × 10^11^). There was strong evidence of accelerated decline in partially vaccinated patients (BF_inclusion_ = 123). Baseline mumps antibody concentrations differed approximately 5.5-fold between groups (1.93 vs. 0.348 IU/mL; *p* = 0.008), and this difference increased to more than sixfold at 12 months (1.34 vs. 0.211 IU/mL; *p* = 0.001).

Overall, these findings suggest heterogeneity in the durability of vaccine-induced immunity, with measles antibodies demonstrating comparatively lower stability than rubella or mumps antibodies. We therefore recommend systematic assessment of vaccination status in all children with IBD. Whenever feasible, catch-up vaccination should be performed prior to the initiation of immunosuppressive therapy, and a personalized approach to immunization strategies should be considered for each patient with IBD.

### Limitations

This study is among the first to prospectively evaluate the longitudinal dynamics of MMR antibodies in children with IBD according to vaccination completeness. Its strengths include the prospective design, the use of validated ELISAs, and the application of Bayesian modeling approaches to assess antibody kinetics. However, several limitations should be acknowledged. First, the absence of a healthy control group precludes direct comparison of the absolute rate of antibody decline with that observed in the general pediatric population. However, this study was specifically designed to evaluate within-cohort heterogeneity in antibody persistence according to vaccination completeness in children with IBD, rather than to compare IBD patients to healthy controls. This design choice was made for several reasons: (1) previous studies have already established that antibody persistence is impaired in children with IBD compared to healthy controls; (2) our primary research question was to identify modifiable risk factors within the IBD population that could inform clinical vaccination strategies; (3) the inclusion of a healthy control group would not have altered the central finding that incomplete vaccination is a major determinant of antibody loss in this vulnerable population. Second, the relatively small sample size may have limited the statistical power of certain subgroup analyses. Vaccination completeness is partially confounded by time since last vaccination, as incompletely vaccinated children typically received only a single dose at 12 months of age. Our survival analysis approach accounts for time since last dose but does not fully disentangle the independent effects of dose number and time. Larger studies with multivariable adjustment are needed to address this complexity. Third, we did not specifically assess the impact of different immunosuppressive therapies on antibody persistence, which may represent an important confounding factor. Despite these limitations, our findings demonstrate a consistent trend toward more rapid loss of vaccine-induced measles, rubella, and mumps antibodies among partially vaccinated patients with IBD. Fourth, our survival analysis is based on cross-sectional baseline data combined with 12-month longitudinal follow-up, rather than true prospective observation from the time of vaccination to antibody loss. This introduces some uncertainty in the exact timing of antibody loss, particularly for patients who were already seronegative at baseline. However, this approach is commonly used in vaccine immunogenicity studies when long-term prospective follow-up from the moment of vaccination is not feasible, and it provides clinically meaningful estimates of antibody durability and the relative impact of vaccination completeness.

## 5. Conclusions

Incomplete MMR vaccination was associated with lower baseline antibody concentrations and reduced persistence of serological protection markers in children with IBD over a 12-month period. Vaccination completeness critically influences both baseline antibody concentrations and the durability of seroprotection over time. Measles antibodies appear particularly vulnerable to waning, even among fully vaccinated patients, underscoring potential gaps in sustained protection within this high-risk population. The markedly earlier loss of seroprotection observed in partially vaccinated patients highlights the clinical consequences of delayed or interrupted immunization schedules. These findings support systematic assessment of vaccination status at the time of IBD diagnosis and emphasize the importance of completing the MMR schedule—preferably prior to the initiation of immunosuppressive therapy These data confirm the importance of assessing vaccination status in the diagnosis of IBD and timely implementation of immunization schedules. However, larger controlled studies are needed to determine whether reduced antibody levels translate into increased clinical risk of vaccine-preventable infections in this population.

## Figures and Tables

**Table 1:** General characteristics of patients.

Characteristic	UC	CD	*p*-Value
IBD phenotypes, n	19	41	n/a
Age at study entry, years, mean ± SD	12.7 ± 3.25	12 ± 3.69	0.457
Age at disease onset, years, mean ± SD	9.36 ± 3.79	10.5 ± 2.6	0.197
Sex, male, n (%)	13 (68.4)	21 (51.2)	0.211
Disease location (Paris classification), n (%)			
E1/L1 n (%)	2 (10.5)	12 (29.3)	n/a
E2/L2 n (%)	4 (21.1)	14 (34.2)	
E3/L3 n (%)	5 (26.3)	15 (36.5)	
E4/L4 n (%)	8 (42.1)	0	
Extraintestinal manifestations, n (%)	1 (5.3)	16 (39)	0.006 *
Unfavorable disease phenotype, n (%)	1 (5.3)	6 (14.6)	0.28
CRP, mg/L, Me (IQR)	4 (0.95–6.5)	4.5 (2.5–6)	0.44
ESR, mm/h, Me (IQR)	11 (7–13.5)	11 (8.75–14.3)	0.500
Fecal calprotectin, µg/g, Me (IQR)	189 (112–218)	202 (133–235)	0.221
Clinical activity score PCDAI, Me (IQR)	n/a	17.5 (12.5–20)	n/a
Clinical activity score PUCAI, Me (IQR)	15 (10–20)	n/a	n/a
Types of therapy			
5-ASA, n (%)	6 (31.6)	7 (17)	n/a
Аza, n (%)	3 (15.8)	12 (29.3)	n/a
Biologic therapy (Inf/Ada), n (%)	10 (52.4)	22 (53.7)	n/a

Notes to Table 1. Data expressed as Me (IQR)—median (interquartile range) or absolute (n) and relative (%) number; mean ± SD—mean ± standard deviation; n/a—no data available. *p* < 0.05 is considered statistically significant. Significant results are marked with an asterisk (*). Abbreviations: UC—ulcerative colitis; CD—Crohn’s disease; PCDAI—Pediatric Crohn’s Disease Activity Index; PUCAI—Pediatric Ulcerative Colitis Activity Index; ESR—erythrocyte sedimentation rate; CRP—C-reactive protein; 5-ASA—5-aminosalicylic acid; Aza—Azathioprine; Ada—Adalimumab; Inf—Infliximab).

**Table 2:** Baseline characteristics according to MMR vaccination completeness.

Characteristic	Complete Vaccination (n = 40)	Incomplete Vaccination (n = 20)	*p*-Value
Age at study entry, years, Median (IQR)	13 (IQR 11–16)	10 (IQR 6.75–14)	0.011 *
Sex, male, n (%)	24 (60)	10 (50)	0.461
IBD, subtype, n (%)			
CD	25 (62.5)	16 (80)	0.170
UC	15 (37.5)	4 (20)	
Age at disease onset, years, Median (IQR)	11 (8–13)	6.5 (5–8.75)	<0.001 *
Time since last MMR dose, months, Median (IQR)	96 (IQR 63.8–125)	105 (IQR 58.3–124)	0.760
Baseline antibody concentrations			
Measles IgG, IUs/mL, Median (IQR)	0.255 (IQR 0.085–0.607)	0.06 (IQR 0.01–0.09)	<0.001 *
Rubella IgG, Is/mL, Median (IQR)	145 (IQR 85.5–170)	35.9 (IQR 8.68–153)	0.016 *
Mumps IgG, OD, Median (IQR)	1.93 (IQR 1.6–2.71)	0.348 (IQR 0.168–1.08)	<0.001 *

Notes to Table 2. Data expressed as Me (IQR)–median (interquartile range) or absolute (n) and relative (%) number. *p* < 0.05 is considered statistically significant. Significant results are marked with an asterisk (*). Abbreviations: UC—ulcerative colitis; CD—Crohn’s disease; IBD—inflammatory bowel disease; MMR—measles–mumps–rubella; IgG—immunoglobulin G; IUs—international units; OD—optical density.

## Data Availability

The data presented in this study are available on request from the corresponding author. The data are not publicly available due to patient privacy concerns and institutional data protection policies.
